# Readability of internet-sourced patient education material related to “labour analgesia”

**DOI:** 10.1097/MD.0000000000008526

**Published:** 2017-11-10

**Authors:** Nilay Boztas, Dilek Omur, Sule Ozbılgın, Gözde Altuntas, Ersan Piskin, Sevda Ozkardesler, Volkan Hanci

**Affiliations:** Dokuz Eylul University, School of Medicine, Department of Anesthesiology and Reanimation, Izmir, Turkey.

**Keywords:** Flesch Reading Ease Score (FRES), Flesch-Kincaid Grade Level (FKGL), Gunning FOG (Frequency of Gobbledygook), labor analgesia, Simple Measure of Gobbledygook (SMOG)

## Abstract

We evaluated the readability of Internet-sourced patient education materials (PEMs) related to “labour analgesia.” In addition to assessing the readability of websites, we aimed to compare commercial, personal, and academic websites.

We used the most popular search engine (http://www.google.com) in our study. The first 100 websites in English that resulted from a search for the key words “labour analgesia” were scanned. Websites that were not in English, graphs, pictures, videos, tables, figures and list formats in the text, all punctuation, the number of words in the text is less than 100 words, feedback forms not related to education, (*Uniform Resource Locator*) URL websites, author information, references, legal disclaimers, and addresses and telephone numbers were excluded.

The texts included in the study were assessed using the Flesch Reading Ease Score (FRES), Flesch-Kincaid Grade Level (FKGL), Simple Measure of Gobbledygook (SMOG), and Gunning Frequency of Gobbledygook (FOG) readability formulae. The number of Latin words within the text was determined.

Analysis of 300-word sections of the texts revealed that the mean FRES was 47.54 ± 12.54 (quite difficult), mean FKGL and SMOG were 11.92 ± 2.59 and 10.57 ± 1.88 years of education, respectively, and mean Gunning FOG was 14.71 ± 2.76 (very difficult). Within 300-word sections, the mean number of Latin words was identified as 16.56 ± 6.37.

In our study, the readability level of Internet-sourced PEM related to “labour analgesia” was identified to be quite high indicating poor readability.

## 1 Introduction

In obstetric analgesia, neuraxial techniques are used to ensure that optimal analgesia has minimal depressant effects on the mother and the baby.^[1]^ During birth, the most common neuraxial techniques are epidural, spinal, and combined spinal-epidural analgesia/anesthesia. Each technique has a variety of advantages and disadvantages and provides temporary pain blockage during birth.^[2]^

Medications used during neuraxial anesthesia include local anesthetics, opioids, and adrenergic agonists. When local anesthetics are combined with opioids, they provide effective analgesia and result in fewer side effects.^[3]^ Of pregnant women, 60% choose neuraxial analgesia during birth.^[4]^

Many patients prefer to meet anesthesiologists face-to-face to obtain information; however, they also wish for written confirmation.^[5]^ Patients may access medical information via Internet-sourced patient education materials (PEMs) and may be able to avoid anxiety and fear when meeting the doctor and save time.^[6]^

Currently, the Internet is one of the most important methods of easily accessing information on many topics including health-related PEMs. There are many articles, websites, and personal blogs with this aim available on the Internet.^[7]^

Many methods have been developed to assess health-related websites such as quality rates, accreditation, and filters of educational tools.^[8]^

Readability provides information on whether a text is easily understood by readers of a certain level by presenting quantitative data related to the text with characteristic properties.^[9]^

There are more than 40 readability analysis formulae, with the Flesch Reading Ease Score (FRES), Flesch-Kincaid Grade Level (FKGL), Simple Measure of Gobbledygook (SMOG), and Gunning (Frequency of Gobbledygook) FOG indices reported as the most commonly used.^[10]^ For calculating these indices, tables, word lists, and figures are not included.

Reading level is generally related to a person's educational level.^[11]^ A variety of health-related organizations (*American Medical Association, National Institute of Health*) have recommended a readability level of 6 or lower for PEM.^[12]^ In America, the health literacy of nearly 80 million people is at the lowest standard.^[13]^ The increase in mobile Internet use is expected to cause a rapid increase in this rate.^[14]^

PageRank is an algorithm developed by Google to rank websites in search engine results. To roughly estimate the importance of a website, the number of links to that page and quality are examined to determine PageRank. This algorithm used by Google to rate pages is not accessible to the public. According to Google, page rating may be classified by the role of key words in the document, the number of visits to the document, and the number and importance of other related websites.^[15]^

Our study aims to assess the readability of Internet-sourced PEM related to “labour analgesia.” In addition to assessing the readability of websites, the authors aimed to compare commercial, personal, and academic websites. With this aim, we assessed the readability of Internet websites using English language.

## 2 Materials and methods

This study was performed in October to December 2013 after receiving permission from Dokuz Eylül University, Faculty of Medicine, Non-Interventional Research Ethics Committee (date: September 26, 2013, protocol no: 2013/35–05, President Dr. Banu Onvural).

The most popular search engine Google (http://www.google.com)^[16]^ was used with the key words “labour analgesia.” The first 100 internet websites related to “labour analgesia” were scanned by 2 independent researchers (Boztas N, Omur D). Commercial websites, personal websites, and official institution websites were noted in the first 100 websites.

Medical journals and websites addressing members of the medical profession were not included in the assessment. The first 100 words at the beginning of the text along with a total of 300 words from the beginning, middle, and end of the text were assessed. The rank values of all the websites were determined and recorded. The texts were copied and saved in Microsoft Office Word 2007 (Microsoft Corporation, Redmond, WA). To evaluate the consistency of results, the research was repeated 3 months later, and results were compared with the previous ones. The readability of the websites was assessed using the FRES, FKGL, SMOG, and Gunning FOG readability formulae obtained from “www.readibility–score.com.”

The FRES formula is 206.835 - (1.015 × mean sentence length) - (84.6 × mean syllable count per word). The FRES score ranges between 0 and 100, with higher values indicating more readability or ease of reading. The scoring is as follows. Scores of 90 to 100: very easy, 80 to 89: quite easy, 70 to 79: easy, 60 to 69: standard, 50 to 59: quite difficult, 30 to 49: difficult and 0 to 29: very complicated.

The texts with scores between 90 and 100 are understood by those with mean 5 years of education, those between 60 and 70 may be understood by those with 8 to 9 years of education, and scores between 0 and 30 may be understood by college graduates.^[15,17,18]^

Although FRES indicates the readability of the text, FKGL is related to the educational level of the individual.^[18]^ FKGL is one of the most commonly used methods for calculating readability; word and sentence length are important, and it indicates educational level. The FKGL formula is (0.39 × mean word count per sentence) + (11.8 × mean syllable count per word) – 15.39. For example, a score of 5 indicates that the text is understood by those with 5 years of education, while a score of 8.6 means the text is understood by those with 8 years of education.^[19]^

In the SMOG formula, a higher number of multi-syllabic words leads to a higher score. Ten consecutive sentences from the beginning, middle, and end of the text are taken, and the number of words with more than 3 syllables are determined. The results of the formula show the readability level of the text. The SMOG formula is 3.1291 + 1.043 × square root [number of multi-syllabic words × (30/ number of sentences)].^[18]^

For Gunning FOG, the sentence length and percentage of multi-syllabic words are important. The Gunning FOG formula is 0.4 × [mean number of words per sentence + 100 (number of multi-syllabic words/ number of words)].^[15]^ The ideal FOG index score is 7 or 8, with a score above 12 accepted as very difficult for most people.^[18]^

The texts obtained from the websites were divided into commercial sites, personal websites, and official organization websites and compared in terms of readability indices, rank values, and number of Latin words within the text.^[15,18]^

A scoring matrix was used to assess the content of PEM. The topics mentioned in the 37 websites included in the study were assessed. The topics in the texts included the following: definition of epidural and spinal analgesia, explanation of how these procedures are performed, effect of neuraxial analgesia on the birth, risks and benefits of the procedure, contraindications, and alternative analgesia methods. Specific risks and side effects included postdural puncture headache, hypotension, dizziness, nausea-vomiting, back pain, lethargy, hemorrhage, infection, shivering, epidural fever, nerve damage, and paralysis.^[20]^

### 2.1 Exclusion criteria

Websites not in English, graphs, pictures, videos, tables, figures and list formats in the text, all punctuation, the number of words in the text is less than 100 words, feedback forms not related to education, URL websites, author information, references, legal disclaimers, and address and telephone numbers were excluded from the assessment to prevent erroneous results.^[7,18]^

### 2.2 Statistical analysis

For statistical analysis, data were uploaded to SPSS Windows 15.0 software (SPSS Inc., Chicago, IL). Continuous values are indicated as mean ± SD, while frequency variables are given as number (n) and percentage (%) (Table 1). For statistical analysis, the Mann–Whitney *U* test was used to compare groups with continuous values such as readability indices and sixth class level. For comparison of frequency variables, the Chi-square or Fisher exact test was used. A *P* value lower than .001 was accepted as a statistically significant difference.

**Table 1:**
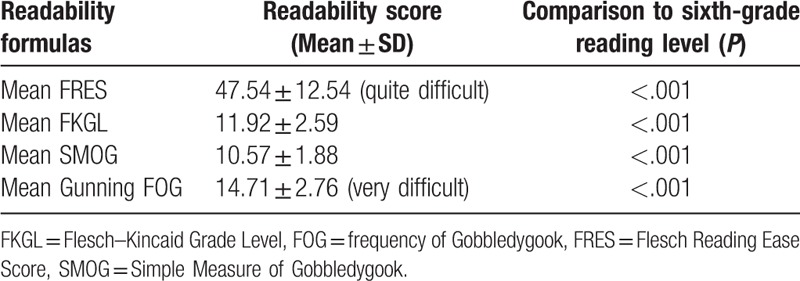
Readability scores of English-language web-based education materials (the first 300 words).

## 3 Results

Of the first 100 websites included in the study, 37 were websites related to informing patients about “labour analgesia”; therefore, 63 websites were excluded. The websites included in the study were classified as commercial websites (n = 22), personal websites (n = 4), and organization websites (n = 11).

Mean readability level according to all readability formulae was identified to be much higher than Level 6 recommended by *American Medical Association* and *the National Institute of Health* (*P* < .05).

The analysis of the first 100 words in the text of the 37 websites revealed mean FRES of 45.80 ± 15.63 (quite difficult), mean FGKL and SMOG of 12.25 ± 3.19 and 12.1 ± 2.47 years of education, respectively, and mean Gunning FOG of 14.99 ± 3.51 (very difficult).

Analysis of the total of 300 words collected from the beginning, middle, and end of the text resulted in mean FRES of 47.54 ± 12.54 (quite difficult), mean FKGL and SMOG of 11.92 ± 2.59 and 10.57 ± 1.88, respectively, and mean Gunning FOG of 14.71 ± 2.76 (very difficult) (Table 1).

Within the sample of 300 words, the mean number of Latin words was identified as 16.56 ± 6.37. Texts from personal websites were found to have significantly higher numbers of Latin words than organization websites. The texts of personal websites were significantly less readable than the commercial websites (Table 2).

**Table 2:**
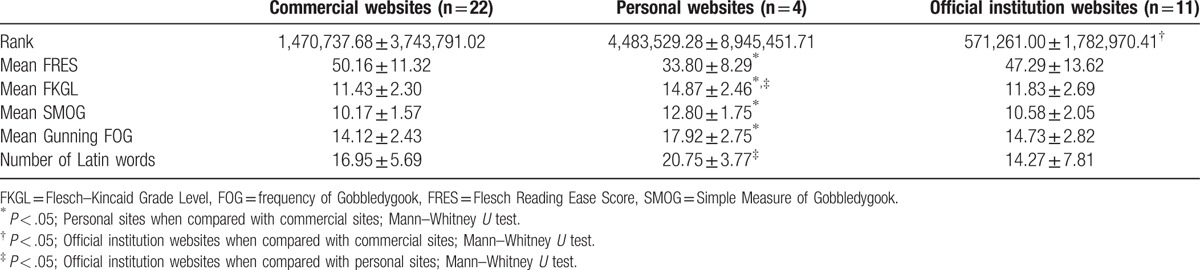
The relationships of web sites between ranks, means of readability grade level, and number of Latin words (the first 300 words).

In our study, when the 300 word sections were investigated, the most common complications were identified as hypotension and postdural puncture headache; in addition, the benefits of epidural procedures and neuraxial analgesia were reported among common topics. Information on risks, side effects, and contraindications of neuraxial analgesia was inadequate (Table 3).

**Table 3:**
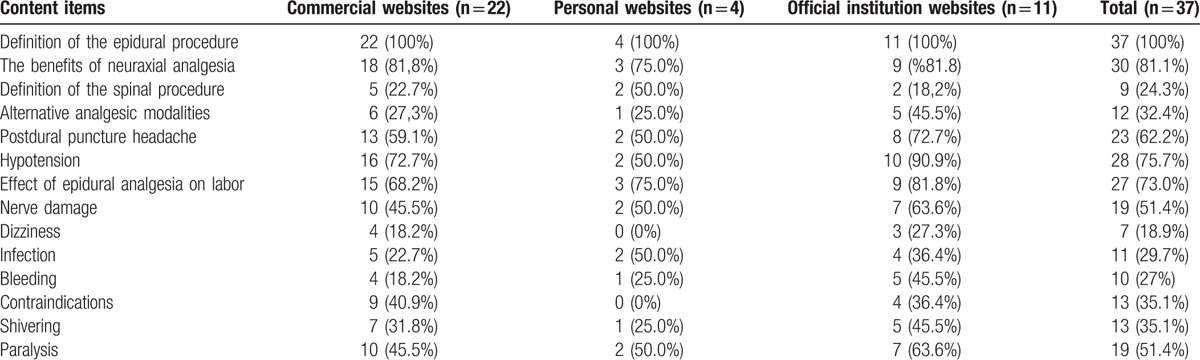
Content analysis of 37 web-based education materials (the first 300 words).

When the correlations between the rank values of the websites; the mean FRES, FKGL, SMOG and Gunning FOG; and number of Latin words were analyzed, no significant correlation was found between any readability index and site rank value (*P* > .05).

## 4 Discussion

Of the first 100 websites in the study, 37 were websites related to patient information about “labour analgesia.” All the websites had readability levels clearly above the recommended 6 years of education level, and our findings are similar to previous studies on the topic.^[18]^ The texts on the personal sites were more difficult to read than the ones on the commercial sites. In our study, the rank values for organization websites were found to be significantly higher than commercial websites. This may indicate that Internet users trust organization websites more. In addition, no significant correlation was determined between rank values of websites and readability indices.

Readability is a very important factor in understanding PEMs. Complex sentences comprised of long words and long sentences may destroy the reader's confidence in learning about a medical situation. It is important to effectively and clearly present accurate information; however, surveys of online health literature have reported texts used thereinto be very technical and complex.^[21]^

For improving the readability of health information, it is reported that sentences should be limited to 8 to 10 words and that simple words should be used instead of complicated medical terminology.^[22]^

Medical terminology is one of the most important factors affecting the readability of a text. Even if the individual's educational level is high, long sentences and unfamiliar words may make the text more difficult to read. In our study, texts on personal websites were found to have significantly high numbers of Latin words compared with organization websites.

This difference may be linked to the medical background of the site authors and insufficient awareness of readability.

There are some disadvantages of using Internet-sourced PEM. Users generally research medical information online on their own initiative; however, those without academic education find it frequently difficult to read and understand medical information.^[23]^ With written material, even if the individual's educational level is high, if they are not accustomed to medical literature, misunderstanding may occur and the individual may cease researching about basic medical care. Therefore, information aiming to educate patients should be clear and understandable.^[17]^ Another disadvantage is that despite this being a consequential decision for the parturient, studies have shown that many patients’ decisions may be driven by a lack of knowledge or by misinformation.^[24,25]^

The readers should be cautious about searching the Internet for information about their health. The readers should consider the possibility of obtaining false or incomplete information. In addition, we recommend that readers choose texts that are more readable. The readability levels of Internet-sourced PEMs and all related sources should be adapted using standard and objective criteria.

Health literacy is defined as the ability of a person to research, obtain, and understand health information and to make the most appropriate health decisions.^[13]^ Low health literacy is correlated with bad health situation, increased hospitalization rates, bad treatment compliance, missed appointments, and increased health spending.^[26]^ This situation was identified more in patients with low socioeconomic level and elderly patients.^[13]^

Cherla et al^[18]^ scanned the first 100 websites belonging to professional organizations, clinical applications, and hospitals related to endoscopic sinus surgery and measured the readability levels of 31 internet-sourced PEM. Similar to our study, they found FKGL to be 10.7, SMOG to be 13.7, Gunning FOG to be 12.4, and FRES to be 47.1.

Svider et al^[17]^ researched the readability levels of Internet websites of academic otolaryngology departments in states in the Central Atlantic region.

With this aim, they used the FKGL, FRES, SMOG, and Gunning FOG indices and identified readability levels of 11 or higher. Svider et al^[17]^ reported the necessity of noncomplex sentences used in short sentences and use of familiar words.

Wang et al^[7]^ assessed the readability levels of 34 articles on the American Orthopedic Academy website and 49 articles on the American Hand Surgery website with the FKGL and Dale–Chall formula and found that the levels were very high, indicating poor readability for the public. They reported that all PEM should be reviewed for readability levels and adapted for public reading.

Sabharwal et al^[19]^ reported that PEM on the American Orthopedic Surgeons Academy website had readability levels above the recommended level (FKGL ≤6). These findings revealed that the readability of the texts on the website needs to be improved. They reported that readability scores are not the only criteria for evaluation of PEM, but that simpler words should be used instead of complex medical language.

For medical knowledge to be understood at optimal levels, comprehension of the informed consent form for patients is an important step. Boztas et al^[27]^ analyzed the readability level of the “anesthesia consent forms” used in universities, Department of Health Education, and research and state hospitals in Turkey. They assessed the first 100 words in the text on the first page of the anesthesia consent form using the Gunning FOG, Flesch-Kincaid, and Atesman Readability formulae. Boztas et al^[27]^ reported that the readability indices of the “anesthesia consent forms” used in Turkish hospitals were very high, indicating low readability. The researchers emphasized that the mean educational level for males is 4 years and 3 years for females in Turkish society. While preparing the anesthesia consent forms, they reported that educational level in the country should be considered and clinicians should carefully handle this medically and legally binding topic.^[27]^

De Oliveira et al^[15]^ used the key word “anesthesia” and evaluated the first 200 websites obtained as Google search results in terms of readability levels. They found that 13 years of education was required to understand the text or the readability was poor.

Patel et al^[20]^ investigated the readability levels of PEMs from 72 English websites and 29 Spanish websites belonging to American medical centers and related to obstetric anesthetic departments. Similar to our study, Patel et al^[20]^ identified that all readability levels according to FKGL, SMOG, and Gunning FOG formulae were well above Grade 6. As with the study by Patel et al,^[20]^ our study reported the most common complications as hypotension and postdural puncture headache and found that the benefits of the epidural procedure and neuraxial anesthesia were the most commonly mentioned topics. Similar to our study, the study by Patel et al^[20]^ found that insufficient information was presented in the texts about the risks, side effects, and contraindications of neuraxial anesthesia.

A limitation of our study is that education and health literacy levels of the patient population were not directly evaluated. The average level of education varies by country. Internet-sourced PEM should match the education level of the population. We used prior studies and comprehension skills of the general public to estimate the average reading skills of patient population seeking “Labour Analgesia.”^[20]^

A second limitation is that the tools we used do not measure the contributions of pictures and diagrams to the comprehension of accompanying text. We can assess the effectiveness of pictures, diagrams, and written text with the Suitability Assessment of Materials.^[28]^ However, this instrument is new, not well validated, and time-consuming.^[29]^ Future studies can explore determining readability scores for large populations, taking into account the effort involved in assessing large populations.

Another limitation is that there is no consensus on the most suitable index for evaluation of readability of Internet-sourced PEM. Each readability index uses a different formula to calculate readability. In this study, the mean readability of PEM remained above the sixth-grade reading level, regardless of which index was used. The FKGL formula can be accessed through Microsoft Office software. FKGL only identifies the number of words in sentences and the number of syllables in words.^[30]^ Syllable counts may not fully indicate readability levels; even medical terms with low syllable counts such as “colon” and “lupus” may not be too readable for individuals who do not know medical terms.^[31]^ The use of the FKGL method is easy; however, this method provides lower estimates of readability measurements than the SMOG and FRES formulae.^[32]^ Understanding of material may be provided by explanatory figures, improved layout, appropriate font type, size, and use of color.^[33]^ FKGL does not evaluate these properties.

In order to avoid this limitation, we used all the 4 indices and compared them. These indices are among the most commonly used formulae and produced results with means above the recommended level.

In conclusion, our study identified that the readability level of internet-sourced PEM related to “labour analgesia” was quite high, indicating poor readability. We recommend that material prepared for patient education and information should particularly be checked for readability indices and should have an appropriate readability level suited to the mean educational level in the relevant country or countries.
